# Case of Ovarian Dropsy, and Hydrothorax

**Published:** 1815-08

**Authors:** 


### *Case of Ovarian Dropsy, and Hydrothorax.*

MISS A. P. at the age of fourteen, of a healthy constitution and comely features, was attacked with severe pains in the right hypogastric region, accompanied with such symptoms, that the attending physician and others in consultation did not hesitate to pronounce her pregnant. Her stomach and habit of body were generally good, though at times deranged, apparently from the severity of the symptoms. In a short time the abdomen became enlarged and lower part of thorax much distended. Superior extremities much emaciated, inferior œdematous. The abdomen became exceeding hard and tense. She continued with progressive enlargement till four or five days previous to dissolution: when loss of appetite, and a laborious and difficult respiration commenced, and death closed the scene.

On the second day permisson was obtained to examine the body. After an incision into the abdomen, there flowed out at least sixty pounds of clear fluid. On further dissection, adhesions of the peritoneum were discovered to a substance filling almost the whole cavity of the abdomen. This preternatural substance extended from the pelvis to the scrobiculus cordis, pressing the liver and stomach against the diaphragm, and that muscle was forced into the thoracic region, so as to lessen its natural space. There were many adhesions sions sions of the omentum and intestines to this substance. The uterus was in a healthy state, with the left fallopian tube and ovarium perfect. The right fallopian tube was distinct and healthy near the uterus; but was soon lost in this enormous formation, which, when separated from the body, was found by accurate weight to be fifty-two pounds. Its removal exposed an enlargement of the mesenteric glands, and considerable inflammation of the intestines. This body, on dissection, was found to be composed of a very great number and variety of cysts, laterally attached to each other. These cysts varied in size, some were not larger than a hazel-nut, others as large as an orange. Their coats, in some parts thick, approaching to scirrhus, in others very thin and soft. On opening these sacs, there was exhibited almost as great a variety of fluid, as number of cysts, some contained aqueous and thin, others slimy fluids, some yellow and green, and others well-digested pus.

The diaphragm was uncommonly thick and rough. On inspecting the thorax, we found the pleura contracted, and the left lobe of the lungs reduced to about one-third of, and the right to about two-thirds of its usual size. Neither of which was otherwise diseased.

The heart and its appendages were healthy. There were judged to be about eight pounds of water in the chest. —*New-England Journal.*

